# Harmonized nitrogen and phosphorus concentrations in the Mississippi/Atchafalaya River Basin from 1980 to 2018

**DOI:** 10.1038/s41597-022-01650-6

**Published:** 2022-08-27

**Authors:** Emma Krasovich, Peiley Lau, Jeanette Tseng, Julia Longmate, Kendon Bell, Solomon Hsiang

**Affiliations:** 1 Global Policy Laboratory, Goldman School of Public Policy, UC Berkeley, Berkeley, CA USA; 2 National Center for Environmental Economics, US Environmental Protection Agency, Washington, D.C., USA; 3 Energy & Resources Group, UC Berkeley, Berkeley, CA USA; 4 Scarlatti, Auckland, New Zealand; 5 Manaaki Whenua – Landcare Research, Auckland, New Zealand; 6 National Bureau of Economic Research, Cambridge, MA USA; 7 Centre for Economic Policy Research, London, UK

**Keywords:** Hydrology, Environmental impact, Environmental monitoring

## Abstract

Water quality monitoring can inform policies that address pollution; however, inconsistent measurement and reporting practices render many observations incomparable across bodies of water, thereby impeding efforts to characterize spatial patterns and long-term trends in pollution. Here, we harmonized 9.2 million publicly available monitor readings from 226 distinct water monitoring authorities spanning the entirety of the Mississippi/Atchafalaya River Basin (MARB) in the United States. We created the Standardized Nitrogen and Phosphorus Dataset (SNAPD), a novel dataset of 4.8 million standardized observations for nitrogen- and phosphorus-containing compounds from 107 thousand sites during 1980–2018. To the best of our knowledge, this dataset represents the largest record of these pollutants in a single river network where measurements can be compared across time and space. We addressed numerous well-documented issues associated with the reporting and interpretation of these water quality data, heretofore unaddressed at this scale, and our approach to water quality data processing can be applied to other nutrient compounds and regions.

## Abstract (summary)

**Table:** 

Measurement(s)	Nitrogen Compound • phosphorus compound
Technology Type(s)	water monitors
Sample Characteristic - Environment	water body • watershed • amount of nitrogen atom in water • water pollution • pollution monitoring • amount of phosphorus in water
Sample Characteristic - Location	river, contiguous United States of America • Mississippi/Atchafalaya River Basin

## Background & Summary

Managing water pollution requires the ability to measure the quantity of pollution in waterways to ensure the effectiveness of pollution mitigation. However, inconsistent water quality reporting practices limit such efforts across many river systems worldwide. Often, there are different sampling and reporting practices amongst local authorities that collect and report water quality measurements along a single river network, or a single authority’s practices at a given sampling site may change over time. In cases where collection and reporting of these measurements are not standardized in advance, the resulting combined dataset may contain inconsistencies that prevent large-scale analysis of spatial patterns and trends in water pollution, since not all observations are comparable to one another. Thus, harmonizing water quality data collected within a river network is a necessary first step towards understanding how pollutants enter and move throughout a river system. Here, we defined harmonization as the process of creating a standardized, quality-controlled dataset that can be used for trend analysis, comparison studies, and modeling.

Incomparability of water quality data poses an acute challenge for managing nonpoint source (NPS) water pollution, which involves the diffuse transport of contaminants into waterways and is predominantly associated with human activities such as agriculture^1,2^. In the United States, nitrogen- and phosphorus-based contaminants associated with fertilizer and livestock waste are the largest source of NPS water pollution and can lead to environmental degradation, ecosystem destruction, and harmful human health outcomes^1–3^. Often, these contaminants originate across expansive regions on land before entering river systems that may be monitored by numerous authorities^4^. To mitigate NPS pollution and its effects, regulators need reliable, standardized water quality data across many different water monitoring authorities to measure the severity of the problem and assess temporal and spatial trends within a river network.

**Fig. 1:**
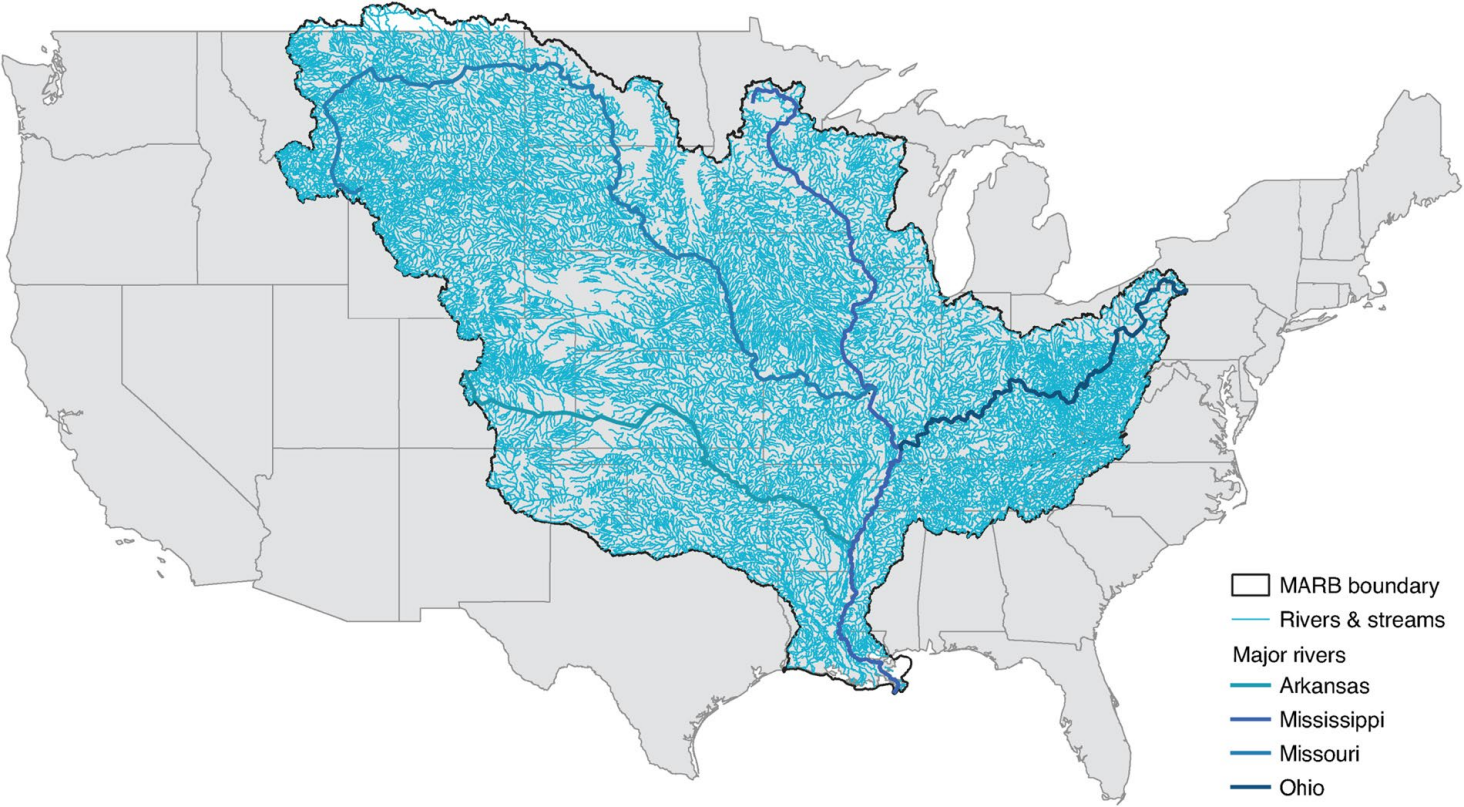
Mississippi/Atchafalaya River Basin and river network^26,27^.

We focused here on harmonizing records of common NPS pollutants throughout the US Mississippi/Atchafalaya River Basin (MARB), which covers 3.2 million square kilometers (roughly 40% of land in the continental US) and crosses 31 state borders, making it the largest river basin in the US and the fourth largest globally (Fig. 1)^2,5^. The MARB has been heavily impacted by NPS water pollutants since at least the 1970s and has suffered from high levels of agricultural runoff. This runoff has resulted in algal blooms, eutrophication, and anoxic conditions that extensively damage ecosystems, reduce the productivity of many marine-dependent industries, and can be toxic to humans and wildlife^6–8^. The vast quantities of NPS water pollution transported by the MARB drain into the Gulf of Mexico, forming a dead zone that covers areas as large as 15,000 square miles^9,10^. The dead zone costs upwards of $USD2.4 billion (in 2018 dollars) every year due to the damage caused to fisheries and marine habitat in the Gulf^11^.

In the United States, hundreds of water monitoring organizations, ranging from local agencies to tribal, state, regional, and federal entities, have collected water quality information on the nation’s 3.5 million miles of waterways, some since the early 1800s^12,13^. In 2012, the United States Geological Survey (USGS), the US Environmental Protection Agency (EPA), and the National Water Quality Monitoring Council jointly established the Water Quality Portal (WQP), a database that serves as the largest access point for publicly-available water quality. The WQP collates data from numerous sources, including the USGS’s National Water Information System (NWIS), the EPA’s STOrage and RETrieval (STORET) Data Warehouse, and the United States Department of Agriculture (USDA) Research Service’s Sustaining The Earth’s Watersheds-Agricultural Research Database System (STEWARDS)^14,15^. At the time of this writing, the WQP contained water quality data dating back to 1892 from over 900 organizations, reporting over 342 million records at more than 900,000 water sampling sites across all 50 states^14^. While the WQP has the potential to be an invaluable resource in assessing water quality issues across the country, the database lacks standardized methods for monitoring, reporting, and storing water quality data^13,15^.

Specifically, some details are critical to accurately interpreting water quality samples, such as the units of measurement (e.g., mg/L or ppm), chemical form of the nutrient (e.g., nitrate or nitrogen), and sample fraction (e.g., filtered or unfiltered), among others^15^. Without standardizing these details, secondary users, such as researchers and policymakers, may not be able to use the data to identify and compare trends across a region where multiple organizations collect water samples. One study found that in a sample of 25 million nutrient records from 488 US organizations measuring water quality data since 1899, 58% could not be interpreted or used due to the lack of standardization across organizations. Recovery of this data “loss” has been valued at $USD12 billion (in 2016 dollars), a number based on US water resource organizations’ investment in collecting and sampling water quality^15^.

In this paper, we retrieved and harmonized WQP water quality data from 136,277 monitoring sites located within the MARB that measure nutrient compounds containing nitrogen (N) and phosphorus (P) between 1980 and 2018. Our objective was to construct a comprehensive sample of observations that were comparable across time and space. Our data collation and harmonization process followed best practices to remove and remedy inconsistencies between and within organizations as detailed by key water quality monitoring organizations, including the EPA, USGS, and USDA^14^. When there was insufficient information to address these inconsistencies, we dropped or flagged these observations.

Here, we detailed our construction of our harmonized water quality dataset, named Standardized Nitrogen and Phosphorus Dataset (SNAPD), which can be used to analyze nonpoint source pollution during a four-decade span in the MARB. Despite the availability of best practices and the known challenges associated with unstandardized water quality data, we were not aware of any other efforts to standardize these data at this scale. To the best of our knowledge, this is the first time that a dataset of standardized N and P water quality concentrations from multiple decades of observations across the MARB will be made publicly available. Our methods can be applied to other water quality monitoring data to address water pollution research questions requiring standardized data from disparate sources. Additionally, our dataset has a number of potential uses, including analysis of both the current status and long-term spatial and temporal trends of river and stream water quality, assessing gaps in monitoring across the MARB, modeling water quality basin-wide to help plan for future monitoring needs, and informing federal rulemaking and permitting. We expect that researchers, water managers, and government agencies at local, state, and federal levels can benefit from access to harmonized MARB water quality data that is comparable across time and space.

## Methods

The Methods section is divided into two subsections: (i) Data source and retrieval, and (ii) Data harmonization.

### Data source and retrieval

We selected and retrieved data for a total of 31 N- and P-based nutrient compounds primarily associated with agricultural runoff from the WQP. For each nutrient compound, we filtered the data to water quality samples measured within the geographic bounds of the MARB and taken between 1980 to 2018. Given these criteria, we retrieved 9,217,921 unique water quality observations from 136,277 water monitoring sites (Table 1).

**Table 1:** Summary of the raw data from the WQP for the 31 selected nutrient compounds associated with NPS pollution within the MARB between 1980–2018.

Nutrient basis	Nutrient name	# of sites	# of samples
Nitrogen-based nutrient compounds	Ammonia*	25,623	390,088
	Ammonia as NH_3_	6,078	57,319
	Ammonia N*	30,620	634,013
	Ammonia N as N	4,907	135,833
	Ammonium as NH_4_	314	1,688
	Kjeldahl N	70,670	1,526,988
	Nitrate*	48,300	793,219
	Nitrate as N	4,825	51,778
	Nitrogen	12,446	248,357
	Nitrogen, mixed forms	21,813	412,803
	Nitrogen nutrient	4,001	75,226
	Organic Nitrogen	22,624	307,805
	Total Ammonia*	30,157	698,963
	Total Kjeldahl N	790	5,142
	Total Kjeldahl N (Organic N plus Nitrate)	3,196	19,864
	Total Nitrogen, mixed forms	3,020	55,133
Phosphorus-based nutrient compounds	Organic Phosphorus	1,088	12,419
	Organic Phosphorus, particulate	1	1
	Orthophosphate*	52,421	1,238,827
	Orthophosphate as P	2,132	63,614
	Orthophosphate as PO_4_	955	7,853
	Phosphate*	24,378	479,954
	Phosphate as P	7,692	104,570
	Phosphate as PO_4_	137	1,300
	Phosphate Phosphorus*	24,372	514,467
	Phosphate Phosphorus as P	7,692	104,567
	Phosphate Phosphorus as PO_4_	129	972
	Phosphorus*	61,091	1,193,311
	Phosphorus, hydrolyzable*	34	764
	Soluble Reactive Phosphorus*	147	4,241
	Total Phosphorus, mixed forms*	7,342	76,842
**Total**		**136,277**	**9,217,921**

Nutrients with an unknown chemical form based on their WQP name are indicated with a *.

### Data harmonization

**Fig. 2:**
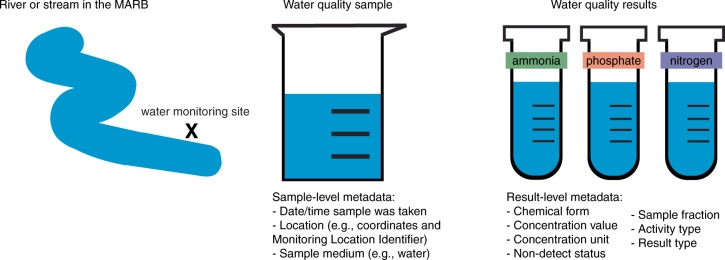
Water quality observation, from sampling to results.

We collated water quality data from 226 organizations. These observations required extensive harmonization of both sample-level and result-level metadata. Sample-level metadata contain a hierarchy of information associated with the collection of a water sample from a water source, such as the site the sample was taken, the date and time, and if the sample was taken from water or soil. A given water sample can then be tested for the presence of multiple nutrient compounds. Result-level metadata contain information specific to the nutrient compound measured in a given sample, such as the concentration of the compound, the filtration status (also referred to as sample fraction), the analytical method used to determine the chemical form, and the detection limit (when applicable), among other information (Fig. 2**)**^15^.

To inform our harmonization process, we used documentation from the Water Quality eXchange (WQX) Nutrient Best Practices Guide to identify and address any data inconsistencies and supplemented as necessary for specific data-quality issues^13–15^. Here, we provided additional background on specific metadata elements requiring harmonization, including nutrient form and nutrient naming, concentration value and units, detection codes and limits, result type, activity type, and sample fraction. We also highlighted the challenges associated with standardizing these metadata and describe how the metadata were harmonized, including any assumptions we made.

As part of our harmonization process, we created two datasets to allow secondary users to choose which best suits their project needs. The first dataset, named SNAPD, is the final output of our harmonization process and has removed observations that did not meet our harmonization criteria. SNAPD contains two data flags: *outlier_flag* which indicates if an observation could be a potential outlier; and *impute_flag* which indicates if an observation was a non-detect and its concentration value was subsequently imputed. We kept these flags in our harmonized dataset as they may be useful for specific analyses.

The second dataset, labeled WQP_to_SNAPD_flagged, retains all of the raw observations that were originally retrieved from the WQP. Instead of dropping data that did not meet our harmonization criteria, we created data flags for each raw observation. These data flags (i.e., “drop” or “keep”) indicate how to harmonize the raw observations following our process, if desired. Because WQP_to_SNAPD_flagged retains all the raw observations and metadata, future users have the option to either contact organizations directly to find missing metadata, accept the decisions and assumptions in our harmonization process, or choose different steps that best suit their research goals.

In this paper, we focus on describing our harmonization process to produce SNAPD, and we also provide additional information in the Data Records section for our secondary dataset, WQP_to_SNAPD_flagged.

#### Creating unique water monitoring sites

We defined a water monitoring site as the unique combination of a Monitoring Location Identifier (MLI) and coordinates that indicate the location where a water quality sample was taken. Where possible, we harmonized coordinates and MLIs as detailed below.

##### Multiple coordinates for the same MLI

Within our dataset, there were 54,578 observations corresponding to 248 MLIs that were flagged as having more than one set of coordinates. All the flagged MLIs had exactly two pairs of coordinates, so for each flagged MLI, we evaluated the Euclidean distance between the coordinate pairs. The distance between the coordinate pairs ranged from under one meter to over 50,000 meters for a given MLI. The same MLI may have different coordinates due to a variety of reasons that would necessitate moving a sampling location a small distance, e.g., river erosion, changing flow patterns, damage to monitoring equipment, or a change in site management from one organization to another.

In many contexts, it is helpful to consolidate coordinates for a single MLI if variations in coordinates do not suggest meaningfully large changes in position. However, large changes in coordinate position may represent material changes in sampling location that may need to be accounted for in any analysis. Thus, if an MLI had a coordinate pair that was greater than 400 meters apart, we assumed that these coordinates were referring to different sites along a river and left both pairs of coordinates for a given MLI as they were. However, when both coordinates for a given MLI were less than 400 meters apart, we harmonized the data such that the MLI only had one unique coordinate. Specifically, we assigned each MLI the corresponding coordinates that first appeared in the data, i.e., we labeled each unique coordinate pair for a given MLI with its respective row number and picked the first row’s coordinates (Table 2: **Step 2**).

**Table 2:** Summary of our data harmonization process to produce the final harmonized dataset, SNAPD.

Harmonization step		Details	Observations affected
Step 0:	Pre-harmonization	Raw data	9,217,921
Step 1:	Organization name	Standardized organization names in instances where there were varied spellings.	568,644
Step 2:	Unique water monitoring sites	Flagged or combined coordinates and Monitoring Location Identifiers (MLIs) where possible such that each water monitoring site was defined as the unique combination of a MLI and coordinate pair.	54,478 (multiple coordinates)
			965,724 (multiple MLIs)
Step 3:	Medium	If the sample was taken in any medium besides water, dropped.	163,356
Step 4:	Date	If an observation was missing a date, dropped.	1,640
Step 5:	Chemical form	If the chemical form of the observation could not be determined, dropped.	1,026,757
Step 6:	Concentration value	If the concentration value was negative, nonsensical (e.g., text instead of a number), or missing and the observation was not indicated to be a non-detect, dropped.	194,579
Step 7:	Concentration units	If concentration units were missing or if they could not be converted to mg/L, dropped.	20,222
Step 8:	Detection Text/codes	If the detection code/text indicated that concentration was not detected due to contamination or other quality control reasons, dropped.	39,868
Step 9:	Sample fraction	If sample fraction was ambiguous or missing, dropped.	340,239
Step 10:	Activity type	If the activity type indicated that the sample was part of a quality control check, dropped.	384,273
Step 11:	Result type	If the result type indicated that the concentration value was estimated, dropped.	130,054
Step 12:	Conversions	Converted nutrients to elemental form (*as P* or *as N*) and converted concentration units to mg/L, where possible.	all
Step 13:	Nutrient renaming	Renamed nutrients to incorporate their sample fraction (e.g., *nitrogen mixed forms unfiltered*, *ammonia filtered*) to ensure comparability of observations.	all
Step 14:	Detection limit approximation	If a detection limit was not provided for a non-detect observation in the raw data, approximated the detection limit (see section on Non-detects, detection codes, and detection limits).	68,533
Step 15:	Non-detect handling	If an observation was indicated as non-detected, imputed concentration value using detection limits (see section on Imputing concentration for non-detects).	1,241,315
		If a nutrient-site-year had 80% or more non-detected observations, flagged observations and left concentration as N/A.	612,918
Step 16:	Outlier flagging	If a given nutrient’s concentration value was above the 99^th^ or below the 1^st^ percentile, flagged as a potential outlier.	131,021
Step 17:	Duplicates	If there were duplicates from multiple concentrations reported for the same site, nutrient, sample fraction, detection status, and date, averaged concentration and indicated the number of observations in the daily average. Note that this also includes time duplicates (see section on Duplicates).	3,191,771
		If there were duplicates due to differently named organizations reporting the same record, chose one organization and assigned to duplicate records.	142,952
		If there were duplicates due to a site measuring both detected and non-detected concentrations on the same date for the same nutrient, averaged concentration and flagged that the average includes an imputed value.	134,848
Step 18:	Nutrients and sample fraction combination	If nutrient sample fractions could be combined to create a more common nutrient (e.g., total phosphorus vs. particulate phosphorus), combined observations where possible (see section on Combining nutrients and sample fractions).	352 (added as new observations)
Step 19:	Data quality	For a given sample, if the filtered nutrient concentration was greater than or equal to the unfiltered nutrient concentration, dropped.	100,050

The number of observations affected by each harmonization step is indicated. Observations may be counted more than once as there may have been more than one harmonization step that affected a given record.

##### Multiple MLIs for the same coordinates

Within our dataset, there were 965,724 observations corresponding to 6,552 unique coordinates that were flagged as having more than one MLI. The number of MLIs associated with a unique coordinate pair ranged from two to 74. MLIs can change if a water monitoring organization starts a new project or the organization responsible for sampling a given site changes. We harmonized the data such that a unique coordinate had only one MLI assigned to it, thereby ensuring that there was a continuous data record of water quality at a given site. We identified observations that had multiple MLIs associated with a unique coordinate and retained the original MLI should a secondary user need this information. We chose the harmonized MLI by assigning row numbers to each unique MLI for a given coordinate and then picked the first row’s MLI (Table 2: **Step 2**).

#### Chemical form

For our dataset, we used a combination of three metadata elements—the nutrient name, concentration units, and analytical method—to identify a nutrient’s *chemical form*. A nutrient’s chemical form indicates whether the concentration of a nutrient compound is reported as a single element, e.g., nitrogen (N), or as a compound, e.g., nitrate (NO_3_). Depending on the chemical form of a nutrient compound, reported concentrations can be interpreted very differently due to the differing mass per unit of volume. For nutrients reported in their elemental nutrient form, concentrations are reported using the elemental weight, which is the concentration of a single atom in a nutrient, e.g., only the N in NO_3_. In contrast, concentrations of nutrients reported in their molecular nutrient form use molecular weight, which is the concentration of the nutrient compound, e.g., nitrate or NO_3_. Assuming the wrong chemical form of a nutrient can result in an incorrect interpretation of the concentration value, thereby introducing error into any calculations^15^.

Where possible, we first recovered a nutrient’s chemical form from the nutrient name itself. For example, a water quality concentration measurement for nitrate might be reported in two ways: elemental form (i.e., nitrate as nitrogen or nitrate as N) or molecular form (i.e., nitrate as nitrate or nitrate as NO_3_). Both naming conventions indicate the nutrient’s chemical form, and therefore the mass of nitrogen that should be accounted for in the respective concentration measurement. For observations in which the nutrient’s chemical form was identified, we harmonized data by converting nutrients to their elemental form, either as N or as P depending on the nutrient compound (Table 2: **Step 5 and Step 12**). However, in some cases, we could not determine the nutrient form based on the nutrient name. As a result, we then relied on either the laboratory method or concentration units to determine the nutrient form. We used a variety of resources, such as the National Environmental Methods Index (NEMI), to identify the chemical form of a compound. For example, a laboratory method for measuring nitrate, known as 4500-NO3-E in NEMI, can be used to determine the concentration of nitrate in water and is reported in units of mg/L as N^16^. Thus, we were able to use metadata that indicated the laboratory method to determine the chemical form that a concentration measurement was reported in. When a nutrient’s chemical form was determined, we converted from molecular to elemental form using conversion factors from the WQX (Table 3)^17^. We removed observations from our harmonized dataset when we could not determine the nutrient’s chemical form from the metadata.

**Table 3:** Conversion factors from molecular to elemental form for all nutrients in our sample requiring conversion^17^.

NUTRIENT NAME	REPORTED MOLECULAR FORM	MULTIPLY BY	DESIRED ELEMENTAL FORM
*AMMONIA*	as NH_3_	0.822	as N
*AMMONIUM*	as NH_4_	0.776	as N
*NITRATE*	as NO_3_	0.225	as N
*ORTHOPHOSPHATE*	as PO_4_	0.326	as P

#### Sample medium

We limited our dataset to observations that were sampled from water. If another type of sample medium was indicated (e.g., soil, air), we dropped these observations (Table 2: **Step 3)**.

#### Concentration and concentration units

In order to compare water quality concentration values across sites and over time, we converted concentration values to a standardized unit of milligrams per liter (mg/L) as N or P depending on the nutrient. To do so, we used *concentration unit* metadata to identify observations that had interpretable units. In some cases, we could not convert observations that had missing concentration units or missing concentration values, and as a result, we removed these observations from our dataset (Table 2: **Step 6, Step 7, and Step 12**). However, missing concentration values could either signify that there was no value associated with a given water quality measurement, and therefore, the observation was truly missing, or that the observation was a *non-detect*. Non-detects were a special case of missing data, which is discussed in the next section.

#### Non-detects, detection codes, and detection limits

Observations with concentrations that lie below a detection limit are a form of censored data known as “non-detects,” since their true concentration value lies somewhere between zero and a given sample processing method’s detection limit. A detection limit is not determined by a chemical constraint inherent in the water sample; rather, each limit is specific to the testing method and equipment used by a laboratory to determine a nutrient’s concentration. Non-detects are reported when a laboratory’s analytical methods cannot distinguish between zero concentration and a positive concentration that is nonzero but below the detection limit^15,17,18^. In general, depending on the monitoring organization, non-detect observations are either reported with concentration values equal to zero, a negative number, or not reported at all. Organizations may also report detection codes alongside non-detect concentration values to indicate relevant details about the analytical method used to determine concentration and its corresponding detection limit^15,17^. Because analytical laboratory methods vary between and within monitoring organizations and across time, there can be many detection limits associated with a given nutrient compound.

For our dataset, we identified non-detects if two conditions were met: (1) if the reported concentration value was zero, negative, or missing and (2) if the *detection code* and *detection limit* metadata indicated that the observation was a non-detect^17^. Next, we created a flag that consolidated the metadata by indicating which observations were non-detects. When the detection code or detection limit metadata indicated that an observation was a non-detect due to contamination or quality control issues with the sample, we dropped these observations (Table 2: **Step 8**).

For our harmonization process, we imputed non-detect observations, and our imputation procedure required that each non-detect observation had an associated detection limit (detailed in the next section). If a detection limit was provided for a non-detected observation, we used that value. In cases where an observation was identified as a non-detect but no detection limit was provided, we approximated a detection limit by assigning a common detection limit based on our data for each nutrient-year (Table 2: **Step 14**). We adopted a conservative approach by assuming that for these observations, non-detects were measured using the least-sensitive methodology that was recorded in our sample across these organizations.

Specifically, when no detection limit was reported for a non-detect observation, we first identified the minimum reported concentration measurement for each organization-nutrient-year combination, among those organizations which report non-detects without a detection limit in that year. We interpreted this minimum concentration to be greater than or equal to the detection limit of the method used by the respective organization. Next, taking this set of minimum concentration values across all the different organizations for the same nutrient-year, we identified the largest value and assigned this as the common detection limit to all non-detect observations that were missing a detection limit for that nutrient-year. In selecting the largest value, we assumed these non-detect observations were measured using the least sensitive method available. This allowed for the detection limit to vary across different nutrient-years since laboratory methods used to measure the concentration could vary across nutrients and over time.

#### Imputing concentration for non-detects

Once we identified non-detect observations that had a detection limit, we imputed their concentration values. We adopted this approach, based on prior analyses^19^, rather than applying alternative substitutions that are sometimes applied, such as: leaving non-detects as missing; dropping them; or substituting in zero, half the detection limit, or the detection limit for missing concentration values^18^. Previous studies have shown these simple substitutions may introduce bias into the data; whereas using statistical imputation to handle non-detects is considered more accurate for computing statistics on data with non-detects^19^. We used a univariate Bayesian imputation method to generate concentration values for any non-detect observation, utilizing a weighted quantile sum regression in the multiple imputation framework^19,20^. The detection limits used for this imputation were based on either the provided or approximated detection limits (see section Non-detects, detection codes, and detection limits for the approximation procedure). Specifically, we employed the *impute.univariate.bayesian.mi* function from the *miWQS* package in the Comprehensive R Archive Network (CRAN)^20^, which uses univariate Bayesian imputation to estimate concentration values for a given site-nutrient-year combination. We only used values from observations sampled at a given site to inform imputed values at that site.

We imputed non-detect values for each set of site-nutrient-year observations in our dataset that had fewer than 80% of its observations identified as non-detects. We chose 80% as the cutoff based on the performance indicators from Hargarten & Wheeler, 2020; however, some analyses identified 50% as a more conservative cutoff^19^. For each of these site-nutrient-year combinations, we constructed ten imputed datasets (K = 10)^21,22^. We averaged the values across the ten imputed datasets to generate one final dataset with one imputed concentration value per non-detected observation. For site-nutrient-year combinations that had more than 80% of their observations flagged as non-detects, we left non-detected values as N/A and created a flag (which we called *impute_flag*), so that secondary users know which observations were non-detects and subsequently imputed (Table 2: **Step 15**).

#### Sample fraction

*Sample fraction* metadata describe the filtration status of water quality observations and may be reported as “dissolved,” “total,” “filtered,” or “unfiltered,” among other categories. This information indicates the composition of particulate (sediment) versus aqueous (liquid) matter of a water quality observation. Depending on the breakdown between particulate and aqueous matter, concentration values for the same nutrient may be very different if the sample fraction is “unfiltered” versus “filtered.”^13,15,17^ In addition, sample fraction metadata are critical to interpretation if naming conventions do not indicate the nutrient form^13,15^.

Currently, to our knowledge, there are no widely adopted reporting standards for sample fraction metadata across organizations, and metadata used to describe the filtration status of a water quality observation for one organization may not be used in the same manner by a different organization. For instance, water monitoring organizations can use the term “total” differently, leading to the misinterpretation of a concentration value. In instances where “total” describes the filtration status of an observation, “total” indicates that a sample contains both the aqueous and particulate portion of one nutrient form (e.g., nitrate) in the concentration value. This would be more clearly described as “unfiltered” sample fraction. In other cases unrelated to filtration status, some organizations use “total” to indicate that a sample contains multiple nutrient chemical forms, such as ammonia (NH_3_) and organic nitrogen (N), and that these nutrient chemical forms are summed to find the total concentration of the elemental form of the nutrient (e.g., total nitrogen). Sample fraction metadata therefore more clearly indicate how to interpret a water quality observation^13,15^.

Concentrations may not be comparable given the same nutrient with different sample fractions. In our dataset, we removed observations if we could not determine both the nutrient form and sample fraction of an observation. However, where possible, we harmonized the nutrient names to include the sample fraction, such as “total nitrogen filtered” or “ammonia unfiltered” (Table 2: **Step 9 and Step 13**). This new categorization allowed us to compare like-nutrient concentrations and sample fractions. In addition, we identified and dropped observations that had a filtered concentration equal to or greater than the unfiltered concentration for a given sample (same site, date, nutrient) (Table 2: **Step 19**)^23^.

#### Activity type

*Activity type* metadata describe the sampling activity that generates a water quality result, such as a field measurement, quality control laboratory sample, routine sample, composite sample, or laboratory replicate, among others. Activity types fell into two categories: (1) activity types that indicate a water quality measurement was taken at a specific water quality monitoring site in the field, and (2) activity types that were not taken at a sampling site in the field and were often associated with laboratory quality controls. We used definitions from the WQP User Guide to identify activity types that did not require a water quality sample to have a specific monitoring location and removed these observations from our dataset (Table 2: **Step 10**)^13,15^.

#### Result type

*Result type* metadata describe the approach used to determine the concentration value from a result. For example, result types can be direct measurements, calculated measurements, or laboratory estimates. We used definitions provided by the WQP User Guide to determine which result types were indicative of a method that might introduce an additional source of error into the data reporting process^13,15^. Specifically, we removed observations with result types that contained the terms “approximation” or “educated guess” from our dataset (Table 2: **Step 11**).

#### Duplicates

Given that the focus of the WQP is on collating discrete samples rather than high-frequency sub-daily samples^23^, we chose to create a daily-level dataset. As part of our harmonization process, we defined and addressed different types of duplicate observations (Table 4) to ensure that each remaining data point was unique to a nutrient, sample fraction, water monitoring site, and date (Table 2: **Step 12 and Step 14**).

**Table 4:** Types of duplicate data in our dataset and the corresponding action taken to harmonize these types of duplicates.

TYPE OF DUPLICATE DATA	HARMONIZATION ACTION
*Multiple concentration values reported for the same site, nutrient, sample fraction, detection status, and date*	Averaged concentration results to be at the daily level for a given site, nutrient, sample fraction, and date, regardless of whether an observation had a timestamp.
*Observations that were reported by different organizations, but were equivalent otherwise*	Combined observations under one organization name.
*Multiple observations with the same nutrient, site, sample fraction, and date, but different detection status (e.g., non-detect vs. observed)*	Averaged observed and imputed non-detect concentrations to be at the daily level for a given site, nutrient, sample fraction, and date. Created a flag to indicate when imputed non-detect and observed concentration values were averaged.

#### Flagging outliers

We identified outliers that were likely from mismeasurement or reporting error, but kept these values in our final dataset, SNAPD. For a given nutrient and sample fraction, we flagged concentration values that were above the 99^th^ percentile or fell below the 1^st^ percentile across all years in our sample, under the variable *outlier_flag* (Table 2: **Step 16**).

#### Combining nutrients and sample fractions

Nitrogen and phosphorus nutrients can sometimes be combined using their sample fractions to determine total nitrogen or total phosphorus on a given date for a given site. Where possible, nutrients were combined using guidance from the National Water Monitoring Council to improve the comparability of observations across time and space^15,23^. For example, we combined dissolved nitrogen (mixed forms) with suspended nitrogen, and we categorized this combination as total nitrogen. Similarly, we aggregated dissolved phosphorus and particulate phosphorus concentration values and categorized the result as total phosphorus^24^. Combinations of nutrients and their sample fractions created an additional 352 observations at sites that had not originally measured the resulting total nutrient on the given date (Table 2: **Step 18**).

## Data Records

We have made our final harmonized dataset, SNAPD, publicly available on HydroShare^25^. The following variables were included in the final harmonized dataset:

- **media**: the medium that the sample was taken in (i.e., water)
- **st_abbr**: the abbreviated name of the US state in which a sample was taken.
- **st_name**: the full name of the US state in which a sample was taken.
- **org_name:** name of the organization or agency responsible for reporting a given water sample. All organization names were standardized, e.g., when there are multiple spellings or abbreviations referring to the same water monitoring organization.
- **N_or_P**: variable to indicate if the nutrient-basis is nitrogen or phosphorus.
- **nutrient_name**: name of the harmonized nutrient compound, e.g., ammonia.
- **sample_fraction**: description of the filtration status of the result, e.g., filtered.
- **nutrient_parameter:** harmonized nutrient name combined with sample fraction, e.g., ammonia_filtered.
- **year:** calendar year the sample was taken.
- **date:** date the sample was taken (format: YYYY-MM-DD).
- **MLI:** abbreviation for Monitoring Location Identifier (MLI), which is a designator used to describe the unique name, number, or code assigned to identify the monitoring location. This variable is an adjusted MLI from the raw data such that each MLI is a unique identifier assigned to a single coordinate pair where water quality samples were collected and results reported (see section **Creating unique water monitoring sites**).
- **conc**: concentration value for a given nutrient parameter (reported or imputed).
- **conc_units**: concentration units reported or converted to be in milligrams per liter (mg/L).
- **outlier_flag**: possible values are “not_flagged_as_outlier,” “potential_outlier,” or “NA”. “not_flagged_as_outlier” indicates that an observation’s concentration was within the 1st and 99th percentiles for a given nutrient; “potential_outlier” indicates that an observation’s concentration was below the 1st or above the 99th percentile for a given nutrient; and “NA” value indicates that the concentration value was missing (because it was a non-detect that was not imputed) so no determination of outlier status was performed.
- **num_obs_per_date:** integer that represents a count of how many concentration measurements were combined via averaging on the same date, for the same site, nutrient, and sample fraction.
- **impute_flag:** possible values are “imputed,” “detected,” or “calculated_by_combining.” “imputed” indicates that the observation was directly identified as a non-detect and concentration values were imputed; “detected” indicates the observation was directly measured; “calculated_by_combining” indicates that the observation was calculated by combining different nutrients and sample fractions.
- **DL:** detection limit for non-detects that was either provided or approximated. This column only contains values for the non-detected concentrations that were imputed.
- **DL_units:** the units for the detection limit value. This column only contains values for the non-detected concentrations that were imputed.
- **x**: longitude coordinate for the unique MLI (site location) in meters (USA Contiguous Albers Equal Area Conic projection, ESRI: 102003).
- **y**: latitude coordinate for the unique MLI (site location) in meters (USA Contiguous Albers Equal Area Conic projection, ESRI: 102003).

**Fig. 3:**
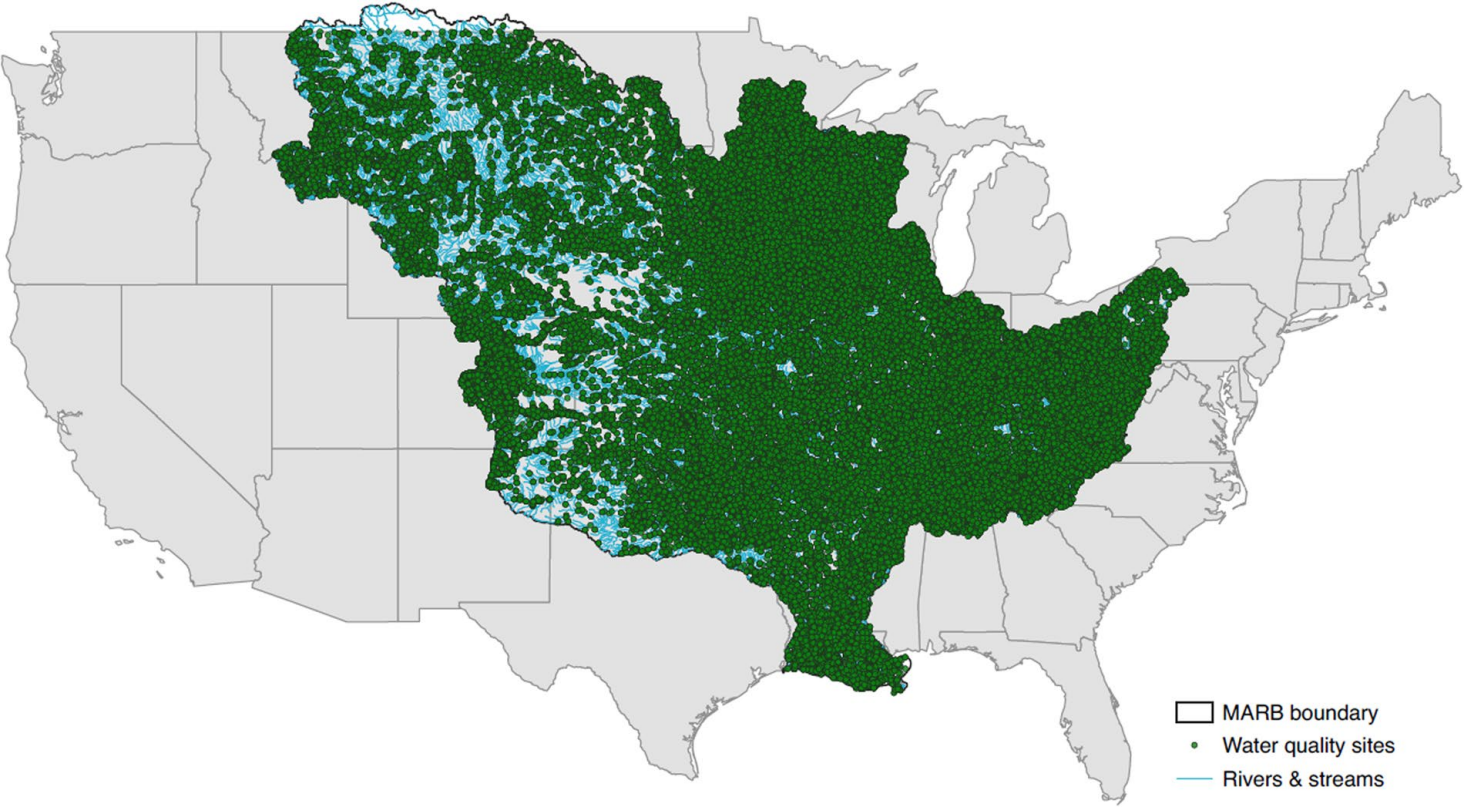
Spatial coverage of SNAPD in the MARB^26,27^.

Table 5 displays the number of sites and observations for each compound we included in the final harmonized dataset, and Fig. 3 maps the corresponding site locations on the MARB network.

**Table 5:** Summary of the final dataset, SNAPD (Standardized Nitrogen and Phosphorus Dataset).

Nutrient basis	Nutrient name	# of sites	# of samples
Nitrogen-based nutrient compounds	Ammonia (filtered)	26,060	275,829
	Ammonia (inorganic)	169	423
	Ammonia (particulate)	67	176
	Ammonia (unfiltered)	49,739	673,734
	Inorganic Nitrogen	98	1,556
	Nitrate (filtered)	20,388	161,368
	Nitrate (inorganic)	491	817
	Nitrate (particulate)	221	329
	Nitrate (unfiltered)	15,215	124,673
	Organic Nitrogen (filtered)	10,978	54,778
	Organic Nitrogen (particulate)	29	62
	Organic Nitrogen (unfiltered)	11,964	164,903
	Kjeldahl Nitrogen (filtered)	10,230	53,300
	Kjeldahl Nitrogen (inorganic)	16	86
	Kjeldahl Nitrogen (particulate)	561	5,019
	Kjeldahl Nitrogen (unfiltered)	55,710	874,093
	Total Nitrogen (filtered)	11,970	61,205
	Total Nitrogen (particulate)	776	11,990
	Total Nitrogen (unfiltered)	21,966	356,456
Phosphorus-based nutrient compounds	Organic Phosphorus (filtered)	159	1,454
	Organic Phosphorus (particulate)	10	13
	Organic Phosphorus (unfiltered)	946	5,282
	Orthophosphate (filtered)	34,373	451,088
	Orthophosphate (inorganic)	170	1,537
	Orthophosphate (organic)	17	38
	Orthophosphate (particulate)	287	656
	Orthophosphate (unfiltered)	30,745	395,815
	Total Phosphorus (filtered)	15,481	164,242
	Total Phosphorus (inorganic)	42	111
	Total Phosphorus (particulate)	203	489
	Total Phosphorus (unfiltered)	61,738	989,986
**TOTAL FOR ALL NUTRIENTS**		**107,149**	**4,831,508**

Our secondary flagged dataset, WQP_to_SNAPD_flagged, contains all raw observations with data flags and is also available on HydroShare^25^. To generate WQP_to_SNAPD_flagged, we followed the same harmonization process as SNAPD, except that we flagged observations instead of dropping them so that secondary users can decide more easily which aspects of our harmonization process are most relevant to their interests. In addition, duplicates were not addressed in this dataset, nor were sample fractions combined to calculate new nutrient concentrations. Secondary users can harmonize WQP_to_SNAPD_flagged by using the data flags as detailed in the variable definitions below. WQP_to_SNAPD_flagged contains the following variables:

- **st_abbr:** the abbreviated name of the US state in which a sample was taken.
- **st_name:** the full name of the US state in which a sample was taken.
- **org_name:** name of the organization or agency responsible for reporting a given water sample. All organization names were standardized, e.g., when there are multiple spellings or abbreviations referring to the same water monitoring organization.
- **N_or_P**: variable to indicate if the nutrient-basis is nitrogen or phosphorus.
- **nutrient_parameter**: harmonized nutrient name combined with sample fraction, e.g., ammonia_filtered.
- **nutrient_handle**: pre-harmonized nutrient name, e.g., ammonia_N_as_N. This variable is unique to WQP_to_SNAPD_flagged.
- **new_MLI**: abbreviation for Monitoring Location Identifier, which is a designator used to describe the unique name, number, or code assigned to identify the monitoring location. This is an adjusted MLI from the raw data such that each MLI is a unique identifier assigned to a single site where water quality samples were collected and results reported. new_MLI corresponds to the variable “MLI” in SNAPD.
- **new_x**: adjusted longitude coordinate for the unique MLI (site location) in meters (USA Contiguous Albers Equal Area Conic projection, ESRI: 102003); this value only differs from the orig_x if coordinates were combined (refer to combine_coord_flag). new_x corresponds to the variable “x” in SNAPD.
- **new_y:** adjusted latitude coordinate for the unique MLI (site location) in meters (USA Contiguous Albers Equal Area Conic projection, ESRI: 102003); this value only differs from the orig_y if coordinates were combined (refer to combine_coord_flag). new_y corresponds to the variable “y” in SNAPD.
- **year**: calendar year the sample was taken.
- **date**: date the sample was taken (format: YYYY-MM-DD).
- **time**: time sample was taken (format: hh:mm:ss), based on a 24-hour timescale.
- **chem_form_flag**: possible values include “chem_form_known,” “chem_form_unknown,” or “NA”. “chem_form_known” indicates that there was sufficient metadata to interpret if the nutrient’s concentration was reported in its elemental or molecular form; “chem_form_unknown” indicates that there was insufficient metadata and the chemical form of the nutrient cannot be determined; “NA” indicates the observation was not flagged because it was already flagged in another harmonization step as “drop.” This variable is unique to WQP_to_SNAPD_flagged.
- **new_conc**: adjusted concentration measure for the nutrient being analyzed. Where possible, this value represents the concentration that has been converted to elemental form and mg/L. new_conc corresponds to the variable “conc” in SNAPD.
- **new_conc_units**: adjusted concentration units reported in mg/L as N or mg/L as P. new_conc_units corresponds to the variable “conc_units” in SNAPD.
- **new_DL**: detection limit value for non-detect observations. This is either reported or approximated when the detection limit is not provided. new_DL corresponds to the variable “DL” in SNAPD.
- **new_DL_units**: adjusted concentration units for detection limits reported in mg/L as N or mg/L as P. new_DL_units corresponds to the variable “DL_units” in SNAPD.
- **ND_flag**: a variable that consolidates all non-detect metadata from other columns. Possible values are “keep,” “ND,” “drop,” or “NA.” “keep” indicates the concentration was detected; “ND” indicates the observation was flagged as a non-detect; “drop” indicates that the observation should be dropped due to either insufficient or poor quality metadata; “NA” indicates the observation was not flagged because it was already flagged in another harmonization step as “drop.” This variable is unique to WQP_to_SNAPD_flagged.
- **impute_flag**: possible values include “dont_impute,” “impute,” or “NA.” “dont_impute” indicates that the concentration value was not imputed either because the concentration value was already provided or because 80% or more of observations for a given nutrient-sample fraction-site-year were non-detects; “impute” indicates that any non-detects at a given nutrient-sample fraction-site-year were imputed; “NA” indicates the observation was not flagged because it was already flagged in another harmonization step as “drop.” impute_flag here is similar to “impute_flag” in SNAPD, but has different possible values because nutrient sample fractions were not combined in WQP_to_SNAPD_flagged.
- **sample_fraction**: description of the filtration status of the result, e.g., filtered.
- **sample_fraction_flag**: possible values are “keep,” “drop,” or “NA.” “keep” indicates that the sample fraction was either directly provided as unfiltered or filtered or could be assumed to represent the same thing (e.g., dissolved or filtered); “drop” indicates that the sample fraction was neither unfiltered nor filtered or any variation thereof (e.g., bed sediment); “NA” indicates the observation was not flagged because it was already flagged in another harmonization step as “drop.” This variable is unique to WQP_to_SNAPD_flagged.
- **result_type:** a brief description of the process which was used in the determination of the concentration value, e.g., actual, estimated, or calculated. This variable was harmonized in SNAPD and is included in WQP_to_SNAPD_flagged to provide secondary users the raw metadata.
- **result_type_flag**: possible values are “keep,” “drop,” and “NA.” “keep” indicates that the result type was provided and of reasonable quality; “drop” indicates that the result type was “estimated” and may introduce error into the reported concentration value; “NA” indicates the observation was not flagged because it was already flagged in another harmonization step as “drop.” This variable is unique to WQP_to_SNAPD_flagged.
- **media:** the medium that the sample was taken in (e.g.,water).
- **media_flag**: possible values are “keep” or “drop.” “keep” indicates that the sample was taken in water; “drop” indicates that the sample was taken in another medium besides water. This variable is unique to WQP_to_SNAPD_flagged.
- **activity_type:** text describing the purpose for the water quality observation, e.g., for water monitoring or laboratory quality control. This variable was harmonized in SNAPD and is included in WQP_to_SNAPD_flagged to provide secondary users the raw metadata.
- **activity_type_flag**: possible values are “keep,” “drop,” and “NA.” “keep” indicates that the activity type was provided and that the sample was taken at a water monitoring site; “drop” indicates that the activity type was for quality control purposes or was not taken at a water monitoring site; “NA” indicates the observation was not flagged because it was already flagged in another harmonization step as “drop.” This variable is unique to WQP_to_SNAPD_flagged.
- **filt2unfilt_flag:** possible values are “keep,” “unfilt conc <  = filt conc,” or “NA”. “keep” indicates that when there were both filtered and unfiltered sample fractions measured on the same date and site, that the filtered concentration measurement was less than the unfiltered concentration in the same sample; “unfilt conc <  = filt conc” indicates that an unfiltered concentration measurement was less than or equal to filtered concentration in the same sample and should be dropped; “NA “indicates that a particular site did not measure both sample fractions for a given nutrient on the same date. This variable is unique to WQP_to_SNAPD_flagged.
- **analytical_method**: the identification number or code assigned by the laboratory method publisher. This variable was harmonized in SNAPD and is included in WQP_to_SNAPD_flagged to provide secondary users the raw metadata.
- **provider**: the name of the database that provided the data to the Water Quality Portal (e.g., WQX, NWIS, STEWARDS). This variable is unique to WQP_to_SNAPD_flagged.
- **orig_conc**: the reported measure of concentration for a given nutrient compound in the raw data. This variable is unique to WQP_to_SNAPD_flagged.
- **orig_conc_units**: the reported concentration units provided in the raw data.
- **conc_flag**: possible values are “keep,” “drop,” and “NA.” “keep” indicates that the concentration value was provided; “drop” indicates that the raw concentration value was negative, zero, or text and was not identified as non-detect; “NA” indicates the observation was not flagged because it was already flagged in another harmonization step as “drop.” This variable is unique to WQP_to_SNAPD_flagged.
- **conc_unit_flag**: possible values are “keep,” “drop,” and “NA.” “keep” indicates that the concentration unit was provided and can be converted to mg/L as N or mg/L as P; “drop” indicates that the concentration unit was either missing or could not be converted to mg/L as N or mg/L as P; “NA” indicates the observation was not flagged because it was already flagged in another harmonization step as “drop.” This variable is unique to WQP_to_SNAPD_flagged.
- **orig_DL_val**: detection limit value provided in the raw data. This variable was harmonized in SNAPD.
- **orig_DL_units**: detection limit concentration units provided in the raw data. This variable was harmonized in SNAPD.
- **DL_code**: a code used to identify any qualifying issues that affected the concentration results. This variable was harmonized in SNAPD.
- **DL_text**: textual description of a result, often indicating non-detect or quality control issues for a given observation. This variable was harmonized in SNAPD.
- **orig_MLI**: MLI is an abbreviation for Monitoring Location Identifier, which is a designator used to describe the unique name, number, or code assigned to identify the monitoring site. This is the original MLI from the raw data download. Note: not all MLIs are unique to a sample location (refer to dup_MLI_flag). This variable was harmonized in SNAPD.
- **dup_MLI_flag**: possible values include “one_MLI” or “dup_MLI.” “one_MLI” indicates that a given coordinate pair (x, y) had only one MLI associated with it; “dup_MLI” indicates that a given coordinate pair (x, y) had more than one MLI associated with it. This variable is unique to WQP_to_SNAPD_flagged.
- **num_MLIs_at_loc**: integer that represents the number of unique MLIs that were associated with a given coordinate pair (x, y). This variable is unique to WQP_to_SNAPD_flagged.
- **orig_x:** raw longitude coordinate for the MLI (site location) in meters (USA Contiguous Albers Equal Area Conic projection, ESRI: 102003). This variable was harmonized in SNAPD.
- **orig_y:** raw latitude coordinate for the MLI (site location) in meters (USA Contiguous Albers Equal Area Conic projection, ESRI: 102003). This variable was harmonized in SNAPD.
- **num_coords_at_loc**: integer that represents the number of unique coordinate pairs (x, y) that were associated with one MLI. This variable is unique to WQP_to_SNAPD_flagged.
- **dup_coords_flag**: possible values include “one_coord_set” or “dup_coords.” “one_coord_set” indicates that a given MLI had one unique coordinate pair (x, y) associated with it; “dup_coords” indicates that a given MLI had multiple unique coordinate (x, y) pairs associated with it. This variable is unique to WQP_to_SNAPD_flagged.
- **combine_coords_flag:** possible values are “combine” or “keep_separate.” “combine” indicates that there were multiple coordinates within 400 m apart associated with one MLI, and these coordinates were consolidated such that a given MLI was assigned a unique coordinate pair; “keep separate” indicates that there were multiple coordinates over 400 m apart associated with one MLI, and no changes were made to these coordinates. This variable is unique to WQP_to_SNAPD_flagged.
- **pct1**: numerical value indicating the (bottom) 1st percentile of all concentration values for a given nutrient. This variable is unique to WQP_to_SNAPD_flagged.
- **pct99**: numerical value indicating the (top) 99th percentile of all concentration values for a given nutrient. This variable is unique to WQP_to_SNAPD_flagged.
- **outlier_flag**: possible values are “not_flagged_as_outlier,” “potential_outlier,” or “NA.” “not_flagged_as_outlier” indicates that an observation’s concentration was within 1st and 99th percentiles for a given nutrient; “potential_outlier” indicates that an observation’s concentration was below the 1st or above the 99th percentile for a given nutrient; “NA” value indicates that this observation was already flagged in another harmonization step as “drop.” This variable corresponds to the “outlier_flag” variable in SNAPD.
- **num_obs_per_date:** integer that indicates the number of observations reported for a given date, MLI, coordinate pair, nutrient, and sample fraction. This variable corresponds to the “outlier_flag” variable in SNAPD.
- **num_orgs_per_obs**: integer that indicates the number of organizations that report the same record for a given date, MLI, coordinate pair, nutrient, concentration, and sample fraction. This variable is unique to WQP_to_SNAPD_flagged.
- **num_nds_per_obs**: integer that indicates the number of detection codes (non-detect or observed) reported for a given date, MLI, coordinate pair, nutrient, and sample fraction. This variable is unique to WQP_to_SNAPD_flagged.
- **num_conc_per_time**: integer that indicates the number of observations reported for a given time, date, MLI, coordinate pair, nutrient, and sample fraction. This variable is unique to WQP_to_SNAPD_flagged.
- **pct_ND**: percentage of observations for a given water monitoring site (unique MLI and coordinate pair combination), nutrient, sample fraction that were non-detected. An “NA” value indicates that this observation was already flagged to be dropped at an earlier harmonization step. This variable is unique to WQP_to_SNAPD_flagged.
- **date_flag**: possible values are “keep” or “drop.” “keep” indicates that the record had a complete date value; “drop” indicates that the record did not have a date associated with it. This variable is unique to WQP_to_SNAPD_flagged.

## Technical Validation

While we had no direct control over the quality of the raw data contained in the WQP, we presented a method that harmonized water quality metadata in ways that were both recommended and necessary to interpret the data and make comparisons across space and time. Our harmonization process followed best practices dictated by the WQX, USGS, and US EPA when available^13,15,17^, in addition to standard data cleaning methods that we detailed above.

However, our dataset has some limitations. Given that we chose to create a daily-level dataset, SNAPD does not offer the detail needed for secondary users to explore water quality trends in one river or stream at a more granular time scale. In addition, we made some assumptions in our harmonization process (e.g., combining coordinates and MLIs, approximating detection limits, and flagging outliers) that other users may choose not to make given their data needs. All assumptions are detailed above and flagged in the intermediate dataset, WQP_to_SNAPD_flagged, thereby allowing secondary users the flexibility to create a version of their own harmonized dataset.

### Code audit

Our harmonization process was audited by an external, independent researcher not associated with this project to verify the logic of our code, check the outcomes of each step, and ensure the replicability of our process and final dataset. We also conducted checks of our data throughout our harmonization process to make sure our data outputs are reasonable, e.g., ensuring that there are no negative concentration values and that filtered concentrations are less than unfiltered concentrations in the same sample. All code is publicly available (**see Code availability**).

### Comparability of compounds

**Fig. 4:**
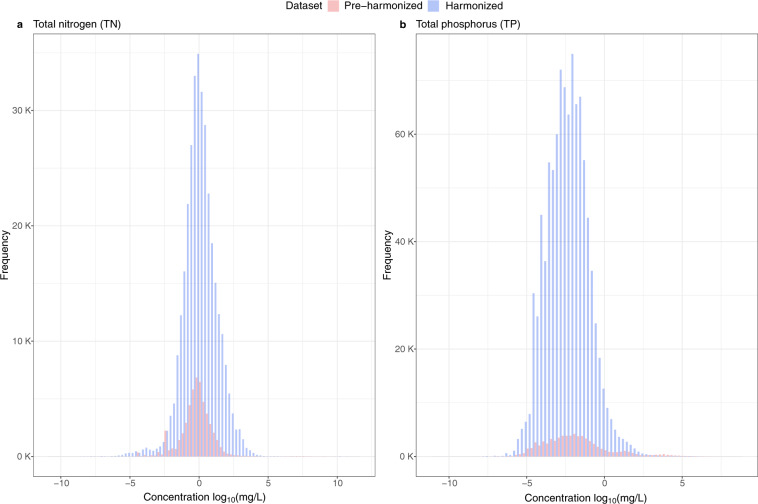
Distribution of the pre-harmonized to harmonized water quality concentration data for all water monitoring sites that measure TN and TP in our retrieved data. The harmonized TN distribution plotted here includes water quality observations that were previously labelled as nitrogen, nitrogen mixed forms, or total nitrogen and are now classified as TN based on our methods. Similarly, water quality observations previously labelled as phosphorus, phosphorus mixed forms, or total phosphorus are now classified as TP based on our methods.

Our harmonization process identified measurements that were comparable in chemical terms, but which would not have been easily comparable in the raw WQP data due to differences in labelling, measurement methods, ambiguous metadata, etc. To demonstrate that our harmonized data improved the number of observations that can be compared to one another, we focused on two nutrients, total nitrogen (TN) and total phosphorus (TP) as examples. We plotted the distribution of the pre-harmonized water quality concentration alongside the harmonized data for TN and TP **(**Fig. 4**)**. For the pre-harmonized data, we only included observations identified as TN or TP in the raw data. To compare concentrations between the pre-harmonized and harmonized datasets, we log-transformed the data. Notably, the harmonized dataset recovered roughly six times as many comparable observations for TN and thirteen times as many for TP than the pre-harmonized data because our harmonization process allowed us to compare standardized observations based on the available metadata.

### Concentration distribution by organization

A contribution of our harmonization process is the standardization of water quality metadata across different reporting standards. This is relevant *across* organizations, which may have different internal standards, but it is also relevant *within* organizations, where standards may change over time, be imprecisely defined, or compliance may be low.

**Fig. 5:**
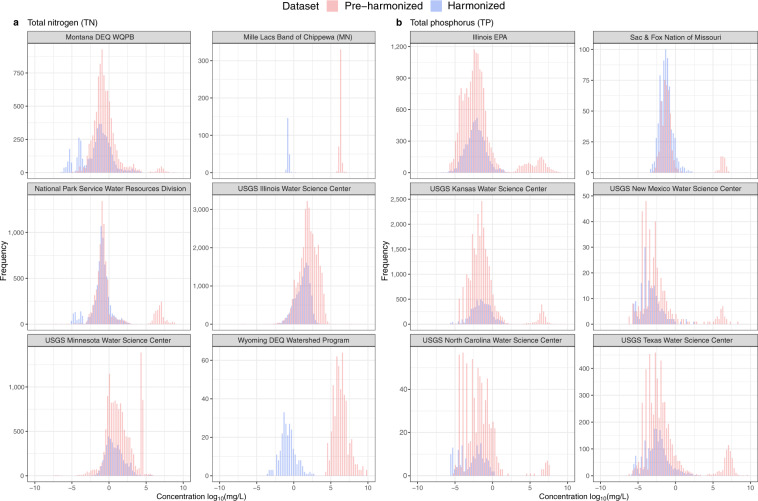
Distribution of the pre-harmonized to harmonized data for selected water monitoring organizations measuring (**a**) total nitrogen and (**b**) total phosphorus. For display, we selected organizations in which our harmonization process impacted both the number of observations and the distribution. We included all raw measurements for either TN or TP that may be harmonized using their metadata. The pre-harmonized distributions included observations measuring total phosphorus, total phosphorus mixed forms, and phosphorus for phosphorus-based nutrients; and nitrogen, nitrogen mixed forms, and total nitrogen mixed forms for nitrogen-based nutrients. Distributions for the harmonized data had fewer observations than those for the pre-harmonized observations because we dropped observations if they could not be harmonized based on metadata.

Here, we plotted the distribution of water quality concentrations (log-transformed) for selected organizations in the MARB that measured TN or TP between 1980–2018 for both the pre-harmonized and harmonized data (Fig. 5). We showcased examples of organizations that displayed distributional shifts in nutrient concentrations following our harmonization process. For this selection of organizations, we observe that the harmonized distribution means are more aligned in comparison to each other than those of the pre-harmonized concentration data. This could suggest that our harmonization process created more comparable nutrient concentrations across organizations.

In addition, the presence of a multimodal distribution could indicate that there exists some internal inconsistency within a given organization. For example, an organization may have different data reporting practices for different nutrient compounds (e.g., nutrient naming and concentration units), and/or their measurement and laboratory methods may have changed over time. Without a secondary source of ground-truth, it is not possible to know with certainty if distribution shifts in nutrient concentration resulted from true changing environmental conditions or from changes in the reporting of environmental conditions. However, cases where our harmonization process impacted the distribution modality within an organization could provide prima facie evidence that inconsistent reporting may have been the source of the multimodal distribution pre-harmonization rather than actual environmental conditions.

While Fig. 5 displays examples of organizations with distributional shifts from pre- to post-harmonization, it is important to note that many organizations not shown here did not demonstrate similar shifts. There may be physical processes that lead to valid multimodal distribution both pre- and post-harmonization, and if an organization’s reporting standards were internally consistent, then we would not expect the modality of specific nutrient concentrations to change.

Figure 5 demonstrates how our harmonization process changed the distribution of nutrient concentrations within organization in different ways, thereby suggesting that the pre-harmonized data contained observations that were likely incomparable both across and within organizations.

### Comparability of units

**Fig. 6:**
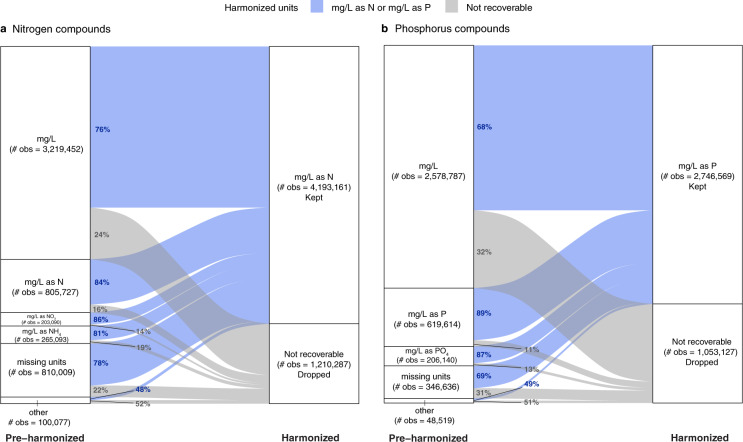
Sankey plots demonstrating the data harmonization process for concentration unit metadata for all nitrogen and phosphorus compounds in our sample. For visualization purposes, we combined concentration units with 50,000 observations or fewer into an “other” category. (**a**) Nitrogen compounds unit harmonization. For nitrogen compounds, the other category includes the following concentration units: #/100 ml, %, % by vol, % by wt, % recovery, cm3/g @stp, cm3/g stp, g/kg, g/m2, mg N/l, mg/g, mg/kg, mg/kg as N, mg/m2 NH4, mgd, MPN, MPN/100 ml, none, NTU, pci/l, ppb, ppm, ueq/l, ug/kg, ug/l, ug/l as N, and umol/l. (**b**) Phosphorus compounds unit harmonization. For phosphorus compounds, the other category includes the following concentration units: #/100 ml, %, cfu/100 ml, g/kg, g/m2, lb/day, mg/g, mg/kg, mg/kg as P, mg/kg PO4, ml/l, mV, none, ppb, ppm, ug/l, and ug/l as P.

Lastly, we examined *concentration units* as one example of metadata that we harmonized. We converted concentration units to “mg/L as N” for all nitrogen compounds and “mg/L as P” for phosphorus compounds. In our sample, there were 32 pre-harmonized concentration units for nitrogen compounds and 21 for phosphorus compounds. These units could not be directly compared to each other. Here, we demonstrated with a Sankey plot the transformation of pre-harmonized concentration units to harmonized units (Fig. 6).

Our harmonization process involved converting and/or scaling concentration data so that water quality observations were comparable. While some concentration units appeared to be commonly reported across water quality organizations in the MARB, these broad categories were not comparable to one another in the pre-harmonized data. All observations must be in the same concentration units to be comparable. Figure 6 highlights the wide variety of concentration units reported in the raw dataset. Harmonizing concentration units was one step of many in our process that needed standardizing. As part of our process, we identified and converted observations to standardized units and chemical form. As a result, we were able to standardize 81% and 75% of pre-harmonized observations for nitrogen and phosphorus compounds, respectively. However, even after harmonizing the concentration units to a standard unit for nitrogen and phosphorus compounds, many observations ultimately were not included in our final dataset due to other metadata quality issues. For instance, some observations lacked sufficient information that would allow us to convert measurements into mg/L, such as “% recovery” or “cm^3^/g.”

## Usage Notes

Our main contribution is the Standardized Nitrogen and Phosphorus Dataset (SNAPD), the first harmonized dataset that allows N and P concentrations to be compared across sites and over time during a four-decade span throughout the Mississippi/Atchafalaya River Basin. This dataset was constructed by combining data from 226 different organizations and transforming all observations into comparable nutrient forms based on heterogenous metadata. When standardization was not possible because the necessary information was not recoverable, observations were removed from the sample. To our knowledge, this is the first dataset that standardized water quality observations across space and time at this scale, for any river basin.

We also provide the intermediate dataset with flags, WQP_to_SNAPD_flagged, to provide secondary users more flexibility in creating a dataset tailored to their needs. WQP_to_SNAPD_flagged allows users to modify our assumptions or refine our harmonization steps, e.g., altering thresholds for outlier detection or imputing non-detects.

It is important to note that our harmonized dataset SNAPD is a subset of the available water quality data stored on the WQP. While our methods are specific to the Mississippi/Atchafalaya River Basin and to our chosen nutrients, other users may apply our harmonization steps to a different region or different water quality variables and keep many of the same steps. We have documented our process in detail and identified key challenges to working with water quality data so that future users can better understand these data and/or make choices in line with their research interests.

## Data Availability

We used R Version 4.0.3, an open-source programming language and environment for statistical computing, to implement our harmonization method. The full harmonization process, starting from the data retrieval to producing the final dataset, is provided in R scripts. All code, data inputs, the final dataset (SNAPD), and the intermediate flagged dataset (WQP_to_SNAPD_flagged) are publicly available on HydroShare here: 10.4211/hs.9547035cf37940eb9b500b7994a378a1^25^.
